# Traditional Chinese medicine formula 01 for nasopharyngeal carcinoma (NPC01) for head & neck cancer and health-related quality of life: a retrospective study

**DOI:** 10.1186/s12906-022-03699-7

**Published:** 2022-08-10

**Authors:** Li Yanwei, He Feng, Wang Xudong, Pan Zhanyu

**Affiliations:** 1 Academy of Medical Engineering and Translational Medicine, Tianjin University, Tianjin, China; 2 Tianjin Key Laboratory of Brain Science and Neural Engineering, Tianjin University, Tianjin, China; 3 Department of Integrative Oncology, Tianjin Medical University Cancer Institute and Hospital and Key Laboratory of Cancer Prevention and Therapy, Tianjin, China

**Keywords:** Traditional Chinese medicine, Head and neck cancer, Quality of life, Symptoms

## Abstract

**Background:**

The traditional Chinese medicine (TCM) formula 01 for nasopharyngeal carcinoma (NPC01) is used in the management of head and neck cancers (HNCs), but whether NPC01 has an impact on the HR-QOL of patients with HNCs is unknown.

**Methods:**

This was a retrospective study of patients with HNCs who were treated between January 2019 and January 2020 at the Head & Neck Cancer Center of Tianjin Medical University Cancer Institute & Hospital. The patients were grouped according to whether they received NPC01 or not (controls). All patients routinely completed the EORTC QLQ-C30 and QLQ-H&N35 modules before and after 3 months of systemic treatment. Health economics were collected.

**Results:**

The patients who received NPC01 were older than the controls (48.6 ± 11.4 vs. 43.4 ± 8.8 years, *P* = 0.004). All other characteristics were comparable between the two groups (all *P* > 0.05). In EORTC QLQ-C30, physical functioning (*P* = 0.03), fatigue (*P* = 0.04), pain (*P* = 0.02), appetite loss (*P* = 0.02), and constipation (*P* = 0.01) scores were improved more in the NPC01 group than in controls. In EORTC H&N35, the scores for pain (*P* = 0.02), swallowing (*P* = 0.01), and dry mouth (*P* = 0.02) were better in the NPC01 group than in controls. The NPC01 cost was 38.94 RMB/patient compared with 12.81 RMB/patient for regular follow-up, but considering insurance coverage, the financial burden was not higher.

**Conclusions:**

The results suggest that NPC01 improves HNCs-related symptoms and HR-QOL.

## Background

Head and neck cancer (HNC) is a heterogeneous collection of malignancies arising in the mucosal linings of the upper aerodigestive tract including the oral cavity, nasopharynx, oropharynx, hypopharynx, and larynx [1–3]. HNC mainly affects people over 50 years old [1] and is more frequent in men than in women [1, 3, 4], with a high prevalence in southern China, northern Africa, and Alaska [2]. HNC is one of the most common cancers, and its incidence is increasing, according to the latest statistics of GLOBOCAN 2020, there were approximately 600,000 new cases worldwide [5]. With the advancements in cancer screening and treatment, the number of HNC survivors is increasing [6, 7]. However, HNC survivors are always associated with dismal outcomes [8, 9]. HNC survivors suffer from disproportionately high levels of psychosocial distress during and after recovery due to the side effects of treatment such as facial disfigurement, swallowing difficulty, and speech dysfunction [10, 11]. Not surprisingly, HNC survivors experience significantly poorer health-related quality of life (HR-QOL) outcomes than survivors of other types of cancer [12].

The traditional Chinese medicine (TCM) formula 01 for nasopharyngeal carcinoma (NPC01) has shown some efficacy in improving the symptoms and signs of HNC [13]. NPC01 is modified from Liang-Ge-San, a traditional Chinese herbal medicine formula derived from the *Taiping Huimin He Ji Ju Fang* (an ancient book of pharmacology of TCM formulae) of the Song Dynasty (AD 960–1279) in China. Liang-Ge-San has been used for centuries in China for the treatment of fever, constipation, pharyngitis, and rhinitis [14]. Some components of NPC01 are known to have immunomodulatory and antiangiogenic effects [13, 15, 16]. The antitumor effect of NPC01 has also been well-addressed [13, 17, 18]. In China, NPC01 has already been used for the maintenance therapy of HNC in clinics, showing favorable efficacy and safety [13, 17]. Nevertheless, whether NPC01 can exert an effect on the HR-QOL results in HNC survivors is unknown. Herein, this study aims to compare the HR-QOL results between HNC patients treated with NPC01 and patients not treated with NPC01, and to further explore the factors leading to the difference of HR-QOL results.

## Methods

### Study design and participants

This study retrospectively analyzed HNC patients treated in the Head & Neck Cancer Center of Tianjin Cancer Hospital from January 2019 to January 2020. This study was approved by the ethics committee of the Tianjin Medical University Cancer Institute & Hospital, Tianjin Medical University (Reference Number: 201902157). The need for informed consent was waived by the committee. The inclusion criteria were: 1) pathologically confirmed HNC and 2) regular follow-up after completing comprehensive treatment including surgery, radiotherapy, and chemotherapy [3]. The exclusion criteria were: 1) loss of follow-up, 2) self-interruption of treatment, 3) treatment-limiting adverse effects, or 4) treatment discontinuation as the physician’s advice.

### Grouping

HNC patients treated with NPC01 were included into the treatment group, while those patients who did not receive NPC01 treatment were included into the control group.

### NPC01 preparation

The ingredients of NPC01 are shown in the Table 1. Every 60 g of NPC01 powder was dissolved in 230 mL of water and was taken once a day for 3 months, and 20 mL of white honey could be added if needed, with warm water delivery service. All ingredients were purchased from Tianjiang Pharmaceutical Co. Ltd. (Jiangyin, Jiangsu, China), one of the six approved manufacturers of Chinese herbal granules in China.

**Table 1:** Composition of NPC01

Latin binomial nomenclature(g)	Catalogue number
*Forsythia suspensa*(120 g)	010301000G
Glycyrrhiza uralensis Fisch (60 g)	010302020Y
Lonicera japonica Thunb (50 g)	01030100SW
Scutellaria baicalensis Georgi (30 g)	010302003D
*Mentha canadensis* Linnaeus (30 g)	010302006A
*Gardenia jasminoides* Ellis (30 g)	010301020R
*Platycodon grandiflorus* (50 g)	010301000D
Gypsum Fibrosum (120 g)	010302000Z
Panax notoginseng (20 g)	0103002002S

### Assessment of quality of life and health economics

All patients routinely received HR-QOL measurement before and after 3 months of systemic treatment. The European Organization for Research and Treatment of Cancer (EORTC) QLQ-C30 and QLQ-H&N35 are commonly used to measure the quality of life in HNC patients [19–21]. To assess health economics, the use of resources, employment status, contacts with community health care services (cancer nurses), and medication costs were routinely collected.

The surveys were conducted by the staff of the Integrated traditional Chinese and Western Medicine Department. Trained psychologists and physicians interviewed patients face-to-face. The questionnaires were administered by a medical doctor. Patient screening was conducted in the outpatient department. The outpatient department had relevant publicity on recruiting research subjects, and patients were informed of the research contents during picking up medicine or waiting in the outpatient department. Each participant was informed of the objectives of this study both in oral and written form. Informed consent from participants was obtained. The interviewers allowed participants to complete the self-assessment questionnaires independently. Participants were encouraged to seek consultation and ask questions as needed.

### Statistical analysis

All data were processed using SPSS 16.0 (IBM, Armonk, NY, USA). Qualitative data were presented as numbers and percentages and analyzed using the chi-square test or Fisher’s exact test. Continuous data were found to be normally distributed and analyzed using the t-test. A value of *P* < 0.05 was considered statistically significant.

## Results

### Patient characteristics

**Fig. 1:**
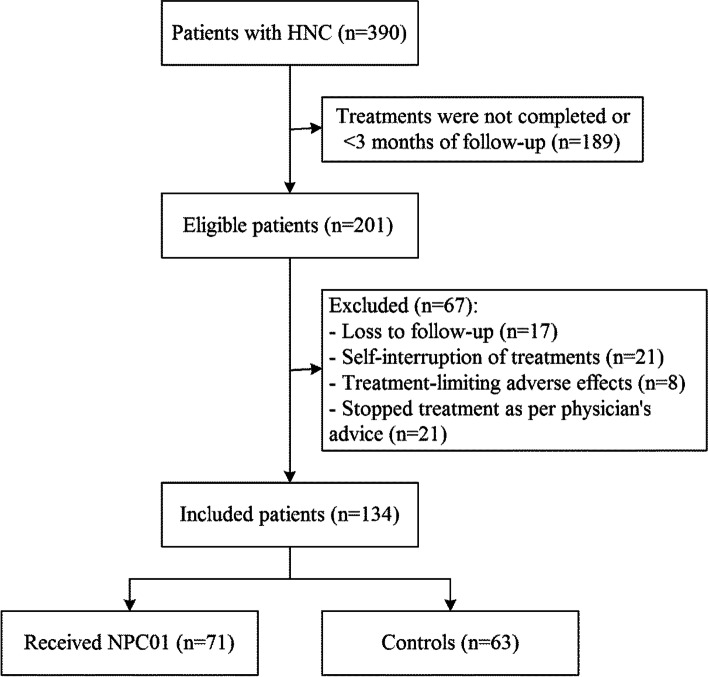
Patient flowchart

Of the 390 HNC patients screened, 71 patients treated with NPC01 for 3 months and 63 patients who received regular treatments were finally included in this study (Fig. 1). The sociodemographic variables and cancer- or treatment-related variables are presented in Table 2. Patients in the NPC01 treatment group were older than those in the control group (48.6 ± 11.4 years old vs. 43.4 ± 8.8 years old, *P* = 0.004). All other characteristics were comparable between the two groups of patients (all *P* > 0.05).

**Table 2:** Characteristics of the patients

Characteristic	NPC01 (*n* = 71)	Controls (*n* = 63)	P
Sex			
Males	25 (32.2)	14 (22.2)	0.099
Females	46 (67.8)	49 (77.8)	0.004
Age, years	48.6 ± 11.4	43.4 ± 8.8	0.325
Education (years)			
< 6	26 (36.6)	17 (27.0)	0.444
6–12	36 (50.7)	35 (55.6)	
> 12	9 (12.7)	11 (17.5)	
Annual family income (RMB)			0.259
< 12,000	23 (32.4)	19 (30.2)	
12,000–240,000	41 (57.8)	42 (66.7)	
≥240,000	7 (9.9)	2 (3.2)	
Marital status			0.606
With spouse	61 (85.9)	56 (88.9)	
Without spouse	10 (14.1)	7 (11.1)	
Employment			0.134
Yes	22 (31.0)	27 (42.9)	
Cancer sites			0.543
Nasopharynx	3 (4.2)		3 (4.8)
Oral cavity	12 (16.9)		9 (14.3)
Oropharynx	21 (29.6)		13 (20.6)
Hypopharynx	20 (28.2)		17 (27.0)
Larynx	15 (21.1)		21 (33.3)
AJCC stage			0.594
I-II	21 (29.6)		14 (22.2)
III-IV	50 (70.4)		49 (77.8)
Treatments			–
Chemotherapy	52 (73.2)		51 (81.0)
Radiation	47 (66.2)		44 (69.8)
Surgery	33 (46.5)		29 (46.0)
Acupuncture	3 (4.2)		7 (11.1)
Other oriental medicine	18 (25.4)		9 (14.3)
Naturopathy	52 (73.2)		51 (81.0)
Herbology or nutrition	10 (14.1)		5 (7.9)
Online support groups	13 (18.3)		21 (33.3)

### Treatment cost

The cost of NPC01 was 38.94 RMB per patient and the cost of regular follow-up was 12.81 RMB per patient. The total cost of each patient in the NPC01 group and control group was 4725 RMB and 2619 RMB respectively (Table 3). We made an exploratory analysis of the cost consequences of participants who had complete cost and outcome data. Since 85% of medical costs was reimbursed by the national medical insurance, NPC01 treatment did not increase the financial burden of participants.

**Table 3:** Costs of health services over 3 months of follow-up

	NPC01(RMB)	Controls (RMB)
Costs	38.94	12.81
General practitioner	0.00	0.00
Community nurse	0.00	0.00
Physiotherapist	0.00	0.00
Speech and language therapist	0.00	0.00
Occupational health therapist	0.00	0.00
Dietician	0.00	0.00
Other healthcare professional	0.00	0.00
Oncology inpatient ward	0.00	0.00
Medical inpatient ward	0.00	0.00
Intensive care inpatient ward	0.00	0.00
Outpatient visits	13.6 ± 11	16.3 ± 25.2
Total costs for 3 months	4725 ± 101	2619 ± 75

The cost of NPC01 was 38.94 RMB per patient and the cost of regular follow-up was 12.81 RMB per patient. The total cost of each patient in the NPC01 group and control group was 4725 RMB and 2619 RMB respectively

### HR-QOL

Significant differences were observed in 5 of the 15 scales in the QLQ-C30 and 3 of the 13 scales in the H&N35 between the two groups (*P* < 0.05). Table 4 shows the HR-QOL indexes. The scores of physical functioning (*P* = 0.03), fatigue (*P* = 0.04), pain (*P* = 0.02), appetite loss (*P* = 0.02), and constipation (*P* = 0.01) scales in the QLQ-C30 questionnaire were significantly improved in the NPC01 group. The scores of pain (*P* = 0.02), swallowing (*P* = 0.01), and dry mouth (*P* = 0.02) scales in the H&N35 questionnaire were also significantly improved in the NPC01 group. After 3 months, the scores of NPC01-treated patients in most scales were increased, but there was no statistically significant difference. Meanwhile, the scores of 5 scales (social functioning, social contact, mouth opening, sticky saliva, and feeling ill) in the NPC01 group were lower than those in the control group, but it was not statistically significant.

**Table 4:** EORTC QLQ-C30 and H&N35 scales over 3 months of follow-up

	Baseline NPC01	3 months NPC01	Change in mean score	Baseline Control	3 months Control	Change in mean score	P
QLQ-C30 Range(0.0–100.0)							
Physical functioning	89.2 (14.1)	82.1 (14.7)	−7.1 (6.2)	83.6 (12.3)	80.7 (14.6)	−2.9 (1.8)	0.03
Role functioning	87.9 (14.9)	85.6 (12.2)	−2.3 (2.5)	87.1 (19.1)	86.6 (19.4)	−0.5 (0.9)	> 0.05
Emotional functioning	79.8 (16.0)	75.6 (10.8)	−4.2 (5.7)	75.6 (19.8)	78.8 (21.3)	−3.2 (3.9)	> 0.05
Cognitive functioning	77.2 (22.7)	77.9 (16.6)	0.7 (0.9)	77.0 (20.1)	77.9 (21.7)	0.9 (1.1)	> 0.05
Social functioning	73.0 (24.0)	76.9 (20.3)	3.9 (5.1)	76.7 (23.7)	78.4 (21.0)	1.7 (2.1)	> 0.05
Fatigue	36.9 (16.8)	32.0 (21.1)	−4.9 (5.3)	32.9 (21.0)	31.9 (11.1)	−1 (1.2)	0.04
Nausea/Vomiting	10.3 (19.2)	7.7 (13.6)	−2.6 (1.8)	10.3 (12.6)	9.2 (15.5)	−1.1 (1.3)	> 0.05
Pain	21.8 (12.6)	16.4 (16.7)	−5.4 (6.7)	19.4 (15.2)	18.7 (17.7)	−0.7 (1.1)	0.02
Dyspnea	16.2 (23.4)	12.1 (18.7)	−4.1 (6.1)	15.9 (18.8)	13.8 (15.8)	−2.1 (5.8)	> 0.05
Insomnia	26.9 (27.5)	23.6 (22.1)	−3.3 (4.5)	26.7 (19.9)	25.4 (25.0)	−1.3 (2.1)	> 0.05
Appetite loss	22.7 (26.7)	13.8 (20.9)	−8.9 (9.1)	20.9 (19.1)	16.4 (21.2)	−4.5 (7.2)	0.02
Constipation	19.8 (15.2)	13.2 (12.5)	−6.6 (5.9)	17.1 (20.4)	18.2 (18.1)	1.1 (1.3)	0.01
Diarrhea	14.8 (21.3)	12.4 (17.8)	−2.4 (2.9)	14.2 (17.6)	13.3 (17.6)	−0.9 (1.1)	> 0.05
Financial problems	24.1 (20.1)	23.1 (14.1)	−1 (1.5)	24.6 (16.7)	23.9 (11.9)	−0.7 (1.2)	> 0.05
EORTC H&N35							
Pain	21.8 (12.6)	16.4 (16.7)	−5.4 (6.7)	19.4 (15.2)	18.7 (17.7)	−0.7 (1.1)	0.02
Swallowing	38.3 (26.6)	23.9 (21.5)	−14.4 (21.3)	35.0 (22.4)	30.9 (21.9)	−4.4 (7.9)	0.01
Senses (taste/smell)	28.1 (19.0)	24.8 (28.1)	−3.3 (4.5)	30.6 (22.7)	27.7 (18.1)	−2.9 (3.9)	> 0.05
Speech	29.4 (26.4)	21.5 (25.7)	−7.9 (8.9)	28.5 (24.0)	22.4 (24.7)	−6.1 (7.7)	> 0.05
Social eating	32.0 (27.5)	28.5 (27.0)	−3.5 (4.2)	28.9 (25.3)	26.1 (18.9)	−2.8 (5.1)	> 0.05
Social contact	23.0 (21.1)	22.7 (24.7)	−0.3 (0.9)	20.9 (20.1)	18.9 (20.3)	−2 (3.4)	> 0.05
Sexuality	NA	NA	NA	NA	NA	NA	
Teeth	44.2 (30.6)	34.0 (29.1)	−10.2 (13.9)	40.9 (23.8)	31.2 (21.0)	−9.7 (14.9)	> 0.05
Opening mouth	36.5 (31.9)	30.2 (20.4)	−6.3 (8.7)	34.9 (22.9)	27.4 (28.1)	−7.5 (9.1)	> 0.05
Dry mouth	56.2 (18.6)	51.7 (19.9)	−4.5 (9.7)	50.1 (19.3)	49.8 (20.1)	−0.3 (1.2)	0.02
Sticky saliva	45.8 (32.3)	36.8 (26.1)	−9 (12.9)	44.7 (23.9)	33.6 (22.9)	−11.1 (13.4)	> 0.05
Coughing	31.0 (24.9)	24.5 (24.0)	−6.5 (8.7)	30.8 (21.9)	27.5 (21.7)	−3.3 (4.1)	> 0.05
Feeling ill	32.3 (26.3)	30.3 (25.0)	−2 (4.7)	34.0 (23.2)	31.1 (23.4)	−2.9 (6.1)	> 0.05

Significant differences were observed in 5 of the 15 scales in the QLQ-C30 and 3 of the 13 scales in the H&N35 between the two groups (P < 0.05)

### Complications

There was no deaths or serious adverse events. Treatment-limiting adverse events were reported in all participants, including headache, dizziness, limb numbness, abdominal distention, stomachache, and diarrhea after drug administration (Table 5), and all these adverse events were grade I. There was no difference in body weight, liver function, kidney function, and hematological indicators between the two groups (data not shown).

**Table 5:** Complications

Adverse events	NPC01	Controls
Headache	2	NA
Dizziness	2	NA
Numbness in hands and feet	1	NA
Stomach Bloating	2	NA
Stomach pain	3	NA
Diarrhea	4	NA
Drowsiness	0	NA

There was no deaths or serious adverse events. Treatment-limiting adverse events were reported in all participants, including headache, dizziness, limb numbness, abdominal distention, stomachache, and diarrhea after drug administration

## Discussion

TCM NPC01 has been extensively applied for the management of HNC [13, 17, 18], but whether NPC01 has an impact on the HR-QOL results of HNC patients remains unknown. Our previously published studies have demonstrated that NPC01 can significantly decrease the expression of angiogenesis-associated factors including hypoxia-inducible factor-1 and vascular endothelial growth factor. The decreased expression of these angiogenesis-associated factors may be due to the inhibition of the phosphoinositide 3-kinase (PI3K)/protein kinase B (Akt)/mammalian target of rapamycin (mTOR) signaling pathway (PI3K/Akt/mTOR). This study compared the HR-QOL results in HNC patients who received NPC01 treatment or not and analyzed the underlying influencing factors. The results suggested that NPC01 improved HNC-related symptoms and HR-QOL results, without increasing the financial burden to the patients. The data of HR-QOL contributes to prognostic risk stratification, in addition to reflecting disease control status and survival rate. In clinical practice, an understanding of HR-QOL helps to integrate patients’ attitudes and treatment goals into the available treatment options. For HNC patients, routine functional tests include swallowing, speech, appearance, pain, activity, and depression. The results reported by patients themselves are usually significantly different from the cognition of doctors. Despite the wide application of NPC01 formula in the clinic, there is still a lack of prospective study on NPC01.

This study aimed to investigate the effect of NPC01 on the HR-QOL outcomes in HNC patients. NPC01 is usually given after surgery, radiotherapy, and chemotherapy as an adjuvant therapy [3] to manage the adverse reactions of conventional treatments. HR-QOL measurement has become a crucial issue for HNC patients, and efforts are now directed towards reducing the impact of the disease and its treatment on patients’ HR-QOL [22–25].

The functional and esthetic organs of HNC patients are inevitably sacrificed following tumor resection. Whether surgery, radiation, chemotherapy, or any combination of them can bring a host of potential complications or adverse results after treatment. Hospital support services for HNC survivors remain inadequate [26]. Currently, the support services for HNC patients in our hospital are mainly focused on those patients at the treatment stage, which are jointly provided by the Cancer Patient Resource Center, advanced practice nurses, nurse navigators, and volunteers. These support resources are rarely used by HNC survivors after treatment, which may be due to the poor access and insufficient referrals [27–29]. In this study, we revealed that NPC01 could improve HR-QOL in HNC survivors by improving physical functioning, fatigue, pain, appetite, constipation, swallowing, and dry mouth. Meanwhile, the scores of social functioning, social contact, mouth opening, sticky saliva, and feeling ill scales in the NPC01 group were lower than those in the control group, but there was no statistical significance. These improved but not statistically significant related indicators might be due to the short follow-up time. In addition, although NPC01 is an oral TCM, patients receiving TCM treatment had regular access to the competent physician and received more attention from the physician than those control patients. Participants considered NPC01 as an effective and harmless therapy. They acknowledged that NPC01 was the basis of optimal and safe treatment. We believed that it was necessary to conduct clinical research to strengthen the recognition and dissemination of NPC01 in Western countries. As this was a retrospective study, the exact use of resources was not evaluated. In addition, it must be noted that the complications of NPC01-treated patients were all grade I, and the patients did not use additional resources to manage these complications.

Although NPC01 has been applied in our cancer center for many years, the formal scientific evaluation of NPC01 is still a brand new field. As the first step in developing a clinical application program of NPC01 for cancer patients, this study is conducted to explore the prospect and experience of NPC01. We also realize that the use of NPC01 is a self-help process with profound cultural deposits, which is related to the traditional Chinese philosophy of life.

HNC risk is strongly associated with low socioeconomic status [28]. Even though the use of NPC01 brings high medical costs, it does not significantly increase the financial burden of patients because TCM is reimbursed at 85% par medical insurances in China. Nevertheless, this study only evaluated patient outcomes at 3 months after treatment, and a long-term prognostic evaluation is needed. Participants also highlighted the long-term positive effects, the benefits of group intervention, and the low cost as important features of TCM.

This study also has some limitations. Firstly, this study uses a retrospective cohort in a single institution. Secondly, the sample size of this study is small. Additional clinical trials are still required to evaluate the benefits of NPC01 [18]. In addition, the research on the evaluation system of Chinese herbal medicine prescription requires further attention and the research methods should also be improved.

In conclusion, NPC01 can improve HR-QOL results in HNC patients. Further research on NPC01 is warranted to determine its exact effectiveness and safety. The findings of this study enrich the relevant knowledge of NPC01 in the treatment of HNC and provide useful information for future clinical research in this field.

## Conclusions

In conclusion, NPC01 can improve HR-QOL in patients with HNC compared with controls with regular follow-up. NPC01 is an effective,economical and safe treatment.

## Data Availability

A summary of the datasets supporting the conclusions of this article is included within the article. Individual patient data sets will not be published; however, cohort data sets may be requested from the corresponding author.

## Abbreviations

TCM: The traditional Chinese medicine
NPC01: Formula 01 for nasopharyngeal carcinoma
HNCs: Head and neck cancers
QOL: Quality of life

## References

[1] Argiris A, Karamouzis MV, Raben D, Ferris RL (2008). Head and neck cancer. Lancet.

[2] Wei WI, Sham JS (2005). Nasopharyngeal carcinoma. Lancet.

[3] Pfister DG, Spencer S, Adelstein D, Adkins D, Darlow SD. Head and neck cancers, version 2.2020, nccn clinical practice guidelines in oncology. J Natl Compr Cancer Netw. 2020:18(7);873–98. 10.6004/jnccn.2020.0031 32634781

[4] Tshering Vogel DW, Zbaeren P, Thoeny HC (2010). Cancer of the oral cavity and oropharynx. Cancer Imaging.

[5] Sung H, Ferlay J, Siegel RL (2021). Global cancer statistics 2020: globocan estimates of incidence and mortality worldwide for 36 cancers in 185 countries. CA Cancer J Clin.

[6] Lo Nigro C, Denaro N, Merlotti A, Merlano M (2017). Head and neck cancer: improving outcomes with a multidisciplinary approach. Cancer Manag Res.

[7] Pulte D, Brenner H (2010). Changes in survival in head and neck cancers in the late 20th and early 21st century: a period analysis. Oncologist.

[8] Ma BB, Hui EP, Wong SC, Tung SY, Yuen KK (2009). Multicenter phase II study of gemcitabine and oxaliplatin in advanced nasopharyngeal carcinoma--correlation with excision repair cross-complementing-1 polymorphisms. Ann Oncol.

[9] Ngan RK, Yiu HH, Lau WH, Yau S, Cheung FY (2002). Combination gemcitabine and cisplatin chemotherapy for metastatic or recurrent nasopharyngeal carcinoma: report of a phase II study. Ann Oncol.

[10] Rogers SN, Semple C, Babb M, Humphris G (2016). Quality of life considerations in head and neck cancer: United Kingdom National Multidisciplinary Guidelines. J Laryngol Otol.

[11] Ojo B, Genden EM, Teng MS, Milbury K, Misiukiewicz KJ (2012). A systematic review of head and neck cancer quality of life assessment instruments. Oral Oncol.

[12] Scott B, Butterworth C, Lowe D, Rogers SN (2008). Factors associated with restricted mouth opening and its relationship to health-related quality of life in patients attending a maxillofacial oncology clinic. Oral Oncol.

[13] Yanwei L, Yinli Y, Pan Z (2018). Traditional herbal formula NPC01 exerts Antiangiogenic effects through inhibiting the PI3K/Akt/mTOR signaling pathway in nasopharyngeal carcinoma cells. Evid Based Complement Alternat Med.

[14] Nagasawa M (1981). [consideration for the relation between a book of prescriptions of Chinese traditional medicine "he ji ju fang" and pharmacopoeia] (Jpn). Yakushigaku Zasshi.

[15] Balap A, Lohidasan S, Sinnathambi A, Mahadik K (2017). Herb-drug interaction of Andrographis paniculata (Nees) extract and andrographolide on pharmacokinetic and pharmacodynamic of naproxen in rats. J Ethnopharmacol.

[16] Tang D, Chen K, Huang L, Li J (2017). Pharmacokinetic properties and drug interactions of apigenin, a natural flavone. Expert Opin Drug Metab Toxicol.

[17] Mao CG, Tao ZZ, Wan LJ, Han JB, Chen Z (2014). The efficacy of traditional Chinese medicine as an adjunctive therapy in nasopharyngeal carcinoma: a systematic review and meta-analysis. J BUON.

[18] Chien CR, Su SY, Cohen L, Lin HW, Lee RT (2012). Use of Chinese medicine among survivors of nasopharyngeal carcinoma in Taiwan: a population-based study. Integr Cancer Ther.

[19] Fayers P, Bottomley A, Group EQoL, Quality of Life U (2002). Quality of life research within the EORTC-the EORTC QLQ-C30. European Organisation for Research and Treatment of Cancer. Eur J Cancer.

[20] Bjordal K, Hammerlid E, Ahlner-Elmqvist M, de Graeff A, Boysen M (1999). Quality of life in head and neck cancer patients: validation of the European Organization for Research and Treatment of Cancer quality of life questionnaire-H&N35. J Clin Oncol.

[21] Chie WC, Hong RL, Lai CC, Ting LL, Hsu MM (2003). Quality of life in patients of nasopharyngeal carcinoma: validation of the Taiwan Chinese version of the EORTC QLQ-C30 and the EORTC QLQ-H&N35. Qual Life Res.

[22] Hammerlid E, Taft C (2001). Health-related quality of life in long-term head and neck cancer survivors: a comparison with general population norms. Br J Cancer.

[23] de Graeff A, de Leeuw JR, Ros WJ, Hordijk GJ, Blijham GH (2000). Long-term quality of life of patients with head and neck cancer. Laryngoscope.

[24] Bjordal K, Ahlner-Elmqvist M, Hammerlid E, Boysen M, Evensen JF (2001). A prospective study of quality of life in head and neck cancer patients. Part II: Longitudinal data. Laryngoscope.

[25] Carvalho AP, McNeely ML, Vital FM (2016). Interventions for preventing and treating trismus in patients with head and neck cancer. Cochrane Database Syst Rev.

[26] Scherpenhuizen A, van Waes AM, Janssen LM, Van Cann EM, Stegeman I (2015). The effect of exercise therapy in head and neck cancer patients in the treatment of radiotherapy-induced trismus: a systematic review. Oral Oncol.

[27] Middel B, Stewart R, Bouma J, van Sonderen E, van den Heuvel WJ (2001). How to validate clinically important change in health-related functional status. Is the magnitude of the effect size consistently related to magnitude of change as indicated by a global question rating?. J Eval Clin Pract.

[28] Eisbruch A, Kim HM, Terrell JE, Marsh LH, Dawson LA (2001). Xerostomia and its predictors following parotid-sparing irradiation of head-and-neck cancer. Int J Radiat Oncol Biol Phys.

[29] Graff P, Lapeyre M, Desandes E, Ortholan C, Bensadoun RJ (2007). Impact of intensity-modulated radiotherapy on health-related quality of life for head and neck cancer patients: matched-pair comparison with conventional radiotherapy. Int J Radiat Oncol Biol Phys.

